# Evidence for lattice-polarization-enhanced field effects at the SrTiO_3_-based heterointerface

**DOI:** 10.1038/srep22418

**Published:** 2016-03-01

**Authors:** Y. Li, H. R. Zhang, Y. Lei, Y. Z. Chen, N. Pryds, Baogen Shen, Jirong Sun

**Affiliations:** 1 Beijing National Laboratory for Condensed Matter Physics and Institute of Physics, Chinese Academy of Sciences, Beijing 100190, P. R. China; 2 Department of Energy Conversion and Storage, Technical University of Denmark, Risø Campus, 4000 Roskilde, Denmark

## Abstract

Electrostatic gating provides a powerful approach to tune the conductivity of the two-dimensional electron liquid between two insulating oxides. For the LaAlO_3_/SrTiO_3_ (LAO/STO) interface, such gating effect could be further enhanced by a strong lattice polarization of STO caused by simultaneous application of gate field and illumination light. Herein, by monitoring the discharging process upon removing the gate field, we give firm evidence for the occurrence of this lattice polarization at the amorphous-LaAlO_3_/SrTiO_3_ interface. Moreover, we find that the lattice polarization is accompanied with a large expansion of the out-of-plane lattice of STO. Photo excitation affects the polarization process by accelerating the field-induced lattice expansion. The present work demonstrates the great potential of combined stimuli in exploring emergent phenomenon at complex oxide interfaces.

The two-dimensional electron liquid (2DEL) at the LaAlO_3_/SrTiO_3_ (LAO/STO) interface has received intensive attention in recent years because of its exotic properties such as 2D superconductivity^1^, 2D ferromagnetism^2^, enhanced Rashba spin-orbital coupling^3^, and strong gating effect^4^,^5^,^6^,^7^,^8^,^9^,^10^,^11^. Among these, the gating effect is particularly interesting. It allows us to tune not only sheet conductance but also interfacial magnetism^12^ as well as Rashba coupling^3^, providing a feasible approach towards emergent phenomena at complex oxide interfaces.

Gate field takes effect through charging/discharging the capacitor formed between 2DEL and gate electrode, tuning carrier density and mobility. The carrier density change, Δ*n*_S_ = εε_0_*V*_G_/*qd*, can be estimated using a simple relation, where ε_0_ and ε are the dielectric constants of vacuum and STO, respectively, V_G_ is gate voltage, *d* is the thickness of STO in the back-gating case, and *q* is the electron charge. Under a V_G_ of 100 V, Δ*n*_S_ is calculated to be ~3 × 10^11^ cm^−2^ near room temperature and ~3 × 10^13^ cm^−2^ at 2 K, adopting the corresponding ε values of ~300 and ~30000 at 300 K and 2 K^13^, respectively. Since the tuned carrier density is still low for the 2DEL at room temperature (~3 × 10^12^ cm^−2^), gating effects on the 2DEL were mainly studied so far below 5 K^4^,^11^, though a systematic investigation above 5 K is obviously necessary. In our previous work^14^, we reported that lattice deformation can be induced, for the amorphous-LaAlO_3_ (a-LAO)/STO interface, by simultaneously applied gate field and visible light, yielding a strong gating effect at room temperature. This discovery provides an approach towards effectively tuning the 2DEL at relatively high temperatures. Although either gating effect or illumination effect has already been reported in the literature^15^,^16^,^17^,^18^,^19^, illumination light and gate field has never been applied simultaneously. The unique light-enhanced gating effect^14^ also raises new questions such as what is the mechanism for the gating effect and how it evolves with temperature as well as what roles the gating field and photo excitation have played in tuning the 2DEL. To answer these questions, in this report, we extended our investigations to cover the whole temperature range from 50 K to 300 K. We found that, with the help of green light, the maximal sheet resistance change, over a wide range of temperature, can be 200-fold larger than the case when only back gating is used. By monitoring the discharging process upon removing the gate field, we give a firm evidence for the occurrence of lattice polarization, which is responsible for the strong responses of the 2DEL. We found that photo excitation affected the lattice polarization by accelerating the process of the field-induced lattice deformation. The present work reveals the great potential of the LAO/STO system for oxide electronics.

## Results and Discussions

### Light illumination-enhanced gating effects

**Figure 1:**
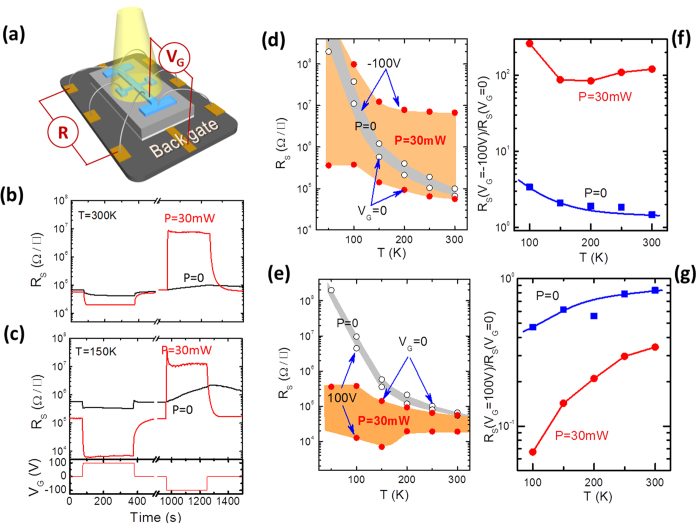
(**a**) A sketch of the experimental setup for sheet resistance measurements. (**b,c**) are sheet resistance of the a-LAO/STO interface corresponding to the on/off-operations of gate field. Labels in the figure denote light power (wavelength λ = 532 nm). (**d,e**) are the sheet resistance as a function of temperature. Light red and grey areas correspond to light on and off, respectively. Labels in the figure mark applied gate voltage. (**f,g**) are the *R*_S_(V_G_)/*R*_S_(0) ratio as a function of temperature. The applied current for the resistance measurements is 1 μA. In all cases the leakage current is found to be lower than 10 nA. All experiments were conducted in a vacuum of 10^−3^ Torr.

The detailed procedures for sample preparation and transport measurements were described in the experimental section. The resulting a-LAO/STO sample is semiconducting, and the corresponding carrier density at room temperature is ~7 × 10^12^ cm^−2 ^ 14. Figure 1 shows two typical resistive responses of a-LAO/STO to electrical stimuli in the presence of light (wavelength = 532 nm), recorded at the temperatures of 150 K and 300 K, respectively. As schemed in Fig. 1a, a gate voltage (V_G_) that switches between −100 V and 100 V was applied to a back gate while the interface was grounded, and sheet resistance (*R*_S_) was recorded in the presence (or absence) of light illumination. In all cases, the leakage current (<10 nA) was much lower than the applied current for resistive measurements (1 μA) (Supplementary information, Fig. 1). Without illumination, as shown in Fig. 1b,c, the gate field triggers the conventional capacitive effect on *R*_S_. Gated by a back gate potential of V_G_ = +/−100 V for 300 s, the *R*_S_(V_G_)/*R*_S_(V_G_ = 0) ratio becomes ~1.5/~0.83 at 300 K and ~2.3/~0.47 at 150 K. As expected, the gating effect alone is not very pronounced. However, when light illumination is applied, the gating effect is dramatically enhanced. When a light of *P = *30 mW (λ = 532 nm) is applied, the negative gate field drives the sheet resistance into a sudden jump with an extremely large resistance. According to Fig. 1d,f, the *R*_S_ is amplified by 120-, 87- and 260-fold at 300, 150 and 100 K, respectively, by a negative gate voltage of −100 V (*R*_S_ is too large to be measured below 100 K). The complex variation of *R*_S_(V_G_ = −100 V)/*R*_S_(V_G_ = 0) with temperature (Fig. 1f) demonstrates the strong temperature dependence of this new gating effect. One thing deserved special attention is the enhancement of the photoelectronic effect at low temperatures. Photo excitation causes a dramatic drop of *R*_S_, while a negative gate field simply helps the interface to restore its initial high resistive state (Fig. 1d). A further unexpected observation is the enhancement of the gating effect under positive biases, though it is not as strong as that under negative biases. As shown in Fig. 1b,c,e,g, a V_G_ of 100 V depresses *R*_S_ by a factor of 3 to 15 when cooling the temperature from 300 to 100 K, respectively.

The enhancement of sheet resistance implies a decrease in carrier density and mobility (μ). In general, a negative gating field prefers to deplete the charge carriers far from interface (which are also charge carriers with a relatively high mobility), resulting in a simultaneous decrease in carrier density and mobility. Hereafter we will focus our attention on *n*_S_ and *R*_S_ since the mobility can be deduced from the formula μ = 1/*en*_S_*R*_S_ if *n*_S_ and *R*_S_ are known, where *e* is electron charge. As proven by our previous work^14^, without illumination, |V_G_| up to 100 V produces no visible changes in the *R*_xy_-*H* slope, indicating that the change in carrier density is small. Aided by an illumination of only 6 mW (532 nm), a V_G_ of −100 V causes the depletion of the carrier density to a value of Δ*n*_S_ ≈ 5.7 × 10^12^ cm^−2^ even at room temperature. This value is about 19 times larger than that one can get from the conventional capacitive effect (Δ*n*_S_ = εε_0_*V*_G_/*dq* ≈ 3 × 10^11^ cm^−2^).

### Lattice deformation-induced lattice polarization

**Figure 2:**
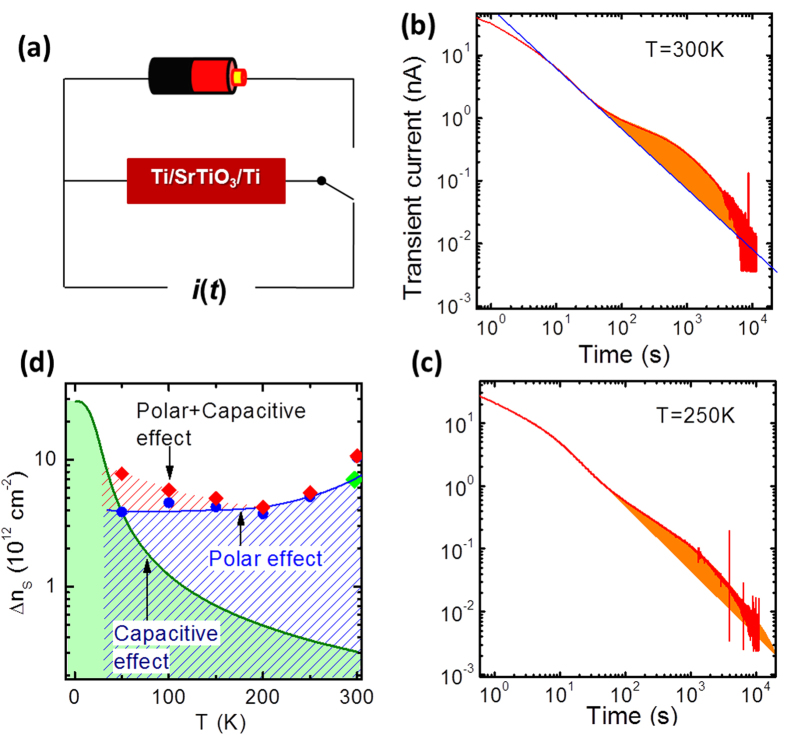
(**a**) A sketch of the experimental setup for transient current measurements. (**b,c**) Discharging current as a function of time, measured at the temperatures of 300 K and 250 K after polling the Ti/STO/Ti capacitor by a V_G_ of 100 V in a laser of 30 mW. Solid line marks the linear background. (**d**) Carrier density changes tuned by the capacitive effect (green line) and lattice polarization (blue symbols), respectively. Red symbols show the expected total carrier density change.

A further issue to be addressed is the underlying mechanism for the enhanced gating effect. It has been reported that, when (001)-STO is sandwiched between two Ti layers, under the influence of gate field the interfacial layer underneath anode will show a lattice expansion^20^. Using Raman spectroscopy, the authors observed new Raman modes accompanying lattice deformation, manifesting a change in crystal symmetry. These phenomena have been suggested to be the consequence of an abnormal lattice polarization due to the non-coincidence of anionic and ionic ion centers^20^. Indeed, if lattice polarization occurs, it will produce an additional tuning knob to the 2DEL. Gate field-induced lattice deformation has been detected by x-ray diffraction (XRD) for our a-LAO/STO (Supplementary information, Fig. 2). However, there is no direct evidences as to whether this lattice deformation results in additional lattice polarization has no direct evidences. Considering the fact that a charge exchange between STO and surroundings will take place in the lattice polarization process, information about lattice polarization could be gained through monitoring discharging current after removing gate field^21^. Since the conductivity of the 2DEL may be inhomogeneous across the interface, as an alternative, we study the discharging process of a TiO_2_-terminated (001)-STO single crystal sandwiched between two metallic electrodes. As schematically shown in Fig. 2a, the Ti/STO/Ti capacitor was first set to a gate voltage of 100 V under light illumination (532 nm), and then short-circuited while the transient current was recorded (at room temperature, the degree for lattice deformation was simultaneously measured by XRD). Figure 2b shows the typical transient current collected at 300 K after a gate voltage of 100 V. The log(*i*)−log(*t*) curve exhibits first a slight downward curvature, a linear decrease, and then a broad upward hump. The former two processes are known as discharging processes for conventional dielectrics. The first one is a transient process immediately following the short-circuiting operation, and the second one is the Curie-von Schweidler process^22^, i.e., log(*i*) linearly decreases with log(*t)* due to a random distribution of discharging barriers. Extrapolating the Curie-von Schweidler law to long time range, we found a hump superimposed on a linear background. This result suggests the occurrence of an extra discharging process. Integrating the broad hump over time, we obtain the total charges exchanged through this additional process (*Q*), and the corresponding carrier density change Δ*n*_S_ =*  Q*/*eS*, where *e* is the electron charge and *S* = 15 mm^2^ is electrode area. A direct calculation shows that the exchanged charges through this process are ~2.4 × 10^−7^ C, corresponding to Δ*n*_S_ ≈1 × 10^13^ cm^−2^. These results confirm the occurrence of lattice polarization. Fascinatingly, the effect of lattice polarization is much stronger than the normal gating effect (it is also the conventional electrical polarization effect of STO; Supplementary information, Note 1) but close to the effect of combined potential and light, suggesting a close relation between enhanced gating effect and lattice polarization.

Indications for lattice polarization are also observed below 300 K. As an example, in Fig. 2c we show the data recorded at 250 K. Compared with the results of 300 K, the main features remain at low temperatures except for a visible downward shift of the entire log(*i*)−log(*t*) curve. The downward shift of the log(*i*)−log(*t*) curve implies a reduction of the discharged charges through the extra discharging process. A simple calculation shows that the carrier density change is ~5.1 × 10^12^ cm^−2^ at 250 K and ~3.9 × 10^12^ cm^−2^ at 50 K. Figure 2d presents the carrier densities depleted by lattice polarization and capacitive effect, respectively. Obviously, in the temperature range well above 50 K, the enhanced gating effect mainly originates from lattice polarization, while in the low temperature range, both lattice polarization and capacitor charging/discharging take effect. It is the combination of these two effects that leads to the complex Δ*n*_S_ -*T* dependence, first decreasing and then increasing upon cooling. Notably, this feature is reminiscent of the non-monotonic variation of *R*_S_(V_G_ = −100 V)/*R*_S_(V_G_ = 0) with temperature. The gating effect under positive V_G_ can be understood in the same scenario. In this case, lattice polarization causes an increase in carrier density and mobility thus a decrease in *R*_S_.

According to the above discussions, a gate voltage of −100 V will tune the carrier density, through polarizing the lattice of STO, by an amount of ~0.4 × 10^13^ to 1 × 10^13^ cm^−2^. Therefore, the 2DELs with a carrier density in this range will be particularly sensitive to lattice polarization. The temperature dependence of lattice polarization is relatively weak, i.e. Δ*n*_S_ is almost constant in a wide temperature range. The slight decrease of lattice polarization at low temperatures may be an indication of weakened lattice deformation; at low temperatures the electro-migration of oxygen vacancies, which is believed to be necessary for lattice deformation, becomes difficult.

### Photo-excitation-accelerated lattice polarization

**Figure 3:**
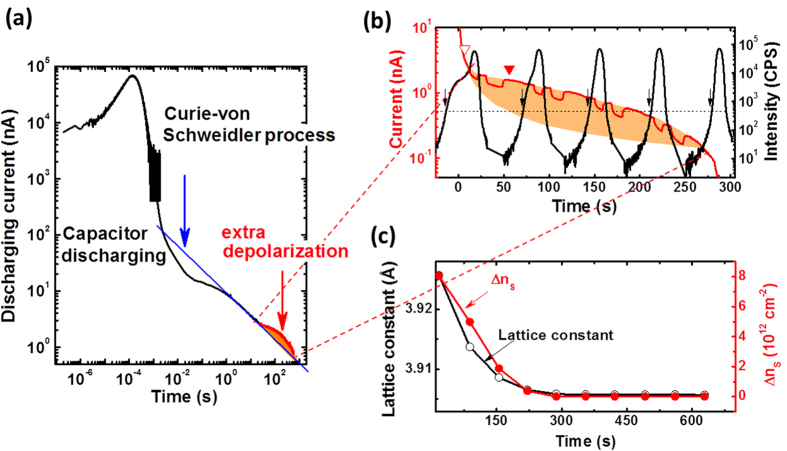
(**a**) Discharging current of the Ti(30 nm)/STO/Ti(30 nm) capacitor polarized by a gate voltage of 100 V in a light of 30 mW (λ = 532 nm), showing when the extra discharging process associated with lattice deformation appears. Shaded area marks the extra discharging process. Straight line is the theoretically calculated Curie-von Schweidler process. (**b**) Transient current and lattice deformation as functions of time, obtained by repeating the θ-2θ scanning of the XRD in the meantime measuring discharging current. Here the θ-2θ spectra are presented along the time axis (scanning speed = 0.0124°/s). Shaded areas mark the extra discharging processes associated with lattice polarization. Open triangle marks the time when the extra discharging process starts, and solid triangle denotes the position where maximal transient current is gained for the extra discharging process. Arrows represent the 2θ angles from which the lattice constants of the deformed interfacial layer are calculated. Dashed line is a guide for the eye. X-ray irradiation promotes the discharging process, leading to a visible increase in transient current. When x-ray is turned on/off or when it is swept through the diffraction peak of STO, where the transient current is depressed probably due to enhanced x-ray reflection, the transient current switches between different values, forming alternatively arranged plateaus and valleys. (**c**) Correspondence between polarization-induced charges and lattice deformation during a discharging process. The former was obtained by integrating the transient current in the shaded area of (**b**) over different time ranges. The lattice constants are calculated from the 2θ angles marked by arrows. Solid lines are guides for the eye. All measurements were performed at room temperature.

A remaining question is how lattice polarization behaves under light. By enhancing the electro-migration of oxygen vacancies, according to our previous work^23^, photo excitation helps the gate field in inducing lattice deformation. In the following we will show that the lattice polarization is always accompanied with lattice deformation. It means that light illumination promotes lattice polarization by accelerating lattice deformation. Figure 3a presents the discharging current as a function of time. The electric process before ~1 s could be ascribed to the discharging of the Ti/STO/Ti capacitor: Adopting the capacitance of ~80 pF and the resistance of ~10 GΩ, the time constant of the *RC* circuit formed by Ti/STO/Ti and an ammeter is ~0.8 s. The following process could be associated with dielectric relaxation and charge trapping/de-trapping, as characterized by the linear log(*i*)−log(*t*) relation. Accompanying this latter process, an extra discharging process occurs in the time window from ~30 s to ~1000 s, signifying lattice depolarization.

Figure 3b is a close view of the process of lattice depolarization. Simultaneously measured XRD spectra are also presented for comparison (here the θ-2θ spectra are unfolded along the time axis; scanning speed = 0.0124°/s). After removing gate field, the crystal structure of STO undergoes a rapid relaxation, returning to unstrained state within 300 s. Accompanying lattice relaxation, an extra discharging process appears (marked by the shaded area in Fig. 3b) and evolves regularly with time. The maximal current discharged through lattice depolarization is ~1 nA, gained at ~56 s (marked by a solid triangle in Fig. 3b). In Fig. 3c we give a comparison of the time dependence of lattice constant and exchanged charge density that is obtained by integrating the shaded area in Fig. 3b from *t*_0_ to *t* (*t*_0_ ≈ 7 s, is the starting time of the extra discharging as marked by an open triangle in Fig. 3b). It is slightly complex to determine the lattice constant of the deformed layer because of the absence of peak splitting in the XRD spectra. Without affecting the general time dependence, we tentatively define 2θ + Δ as the peak position for deformed layer, where 2θ is the 002 peak position of unstrained STO and Δ is the low angle shift caused by lattice expansion (Supplementary information, Note 2). As shown in Fig. 3c, lattice relaxation is most rapid in the first 150 s after the removal of the gate field, slows down afterwards, and finishes within 300 s. Remarkably, the discharged charge quantity shows exactly the same tendency, changing from fast to slow with time. When tuning lattice expansion by gate voltage, we observed a similar relation between lattice constant and tuned carrier density (Supplementary information, Fig. 3). These results confirm the simultaneous occurrence of lattice polarization and lattice deformation. Based on our previous work on illumination effect^23^, we can conclude that photo excitation accelerates lattice polarization by affecting the field-induced lattice expansion.

In summary, we found a gate field-induced lattice polarization, which leads to strong tuning of the 2DEL at the a-LAO/STO interface below 300 K. A relation between lattice polarization and lattice deformation and their relation to the carrier density at the interfaces is established. We found that light illumination affects lattice polarization by enhancing the field-induced lattice deformation. The principle proven here can be extended to other provskite oxides, opening avenues towards emergent phenomena at complex oxide interfaces.

## Methods

### Sample fabrication

The a-LAO/STO 2DEL was prepared by depositing a a-LAO layer at room temperature, ~12 nm in thickness, on a TiO_2_-terminated (001)-STO substrate (3 × 5 × 0.5 mm^3^) using the pulsed laser ablation technique (wavelength = 248 nm). In the deposition process, the substrate was kept at ambient temperature and the oxygen pressure at 10^−3^ mbar. The fluence and the repetition rate were kept constant at 1.5 Jcm^−2^ and 1 Hz, respectively. The target-substrate distance was 4.5 cm. A shadow mask was employed to get Hall-bar-shaped samples. Detailed procedures for sample preparation can be found in ref. 24. The Ti/STO/Ti sandwiched samples for transient current measurements were obtained by depositing two 30-nm-thick Ti layers on the top and back side of a (001)-STO substrate (3 × 5 × 0.5 mm^3^, doubly polished), respectively, using magnetron sputtering in an Ar atmosphere of the pressure 5 × 10^−3^ mbar at room temperature. The Ti electrode covers the whole surface of the STO substrate. Since it is only 30 nm in thickness, laser light can still pass through it without obvious reflection from the metal layer.

### Measurements

The resistive measurements were performed in a home-made system that has a vacuum chamber with a window for incident laser and a cycling cryogenic refrigerator for temperature controlling. Ultrasonic Al wire bonding (20 μm in diameter) was used for electric connection. Four-probe technique was adopted for resistive measurements. The four welding spots were well aligned, with a separation between neighbouring spots of ~0.4 mm. The formula of *R*_S_ ≈ (*L*/*W*)*R* was adopted for the convention of four-probe resistance to calculate the sheet resistance, where *L* and *W* are respectively the long and wide dimensions of the measured plane. Transverse electrical field was applied to STO through an Ag electrode underneath STO, and the a-LAO/STO interface was grounded. The applied current for resistance measurements was 1 μA. Lasers with a wavelength of 532 nm was adopted in the present experiments. The spot size of the light was ~0.4 mm in diameter, focusing on the space between two inner Al wires. All resistive measurements were conducted in a vacuum chamber with a base pressure of 10^−3^ Torr.

Transient current of Ti(30 nm)/STO/Ti(30 nm) was measured by two-point technique in the following procedure: The sample was set to a stably charged state by gating the sample for a duration of 1 hour with the help of a laser beam of 100 mW and 532 nm, and then discharging current was recorded as a function of time just after two electrodes being short-circuited. A Tektronix oscilloscope (TDS3052C) and a Keithley SourceMeter (2611) were employed to acquire discharging current in the short and long time windows, respectively.

The effect of combined gate field and light illumination on the structure of Ti(30 nm)/SrTiO_3_/Ti(30 nm) was measured by a Bruker diffractometer (D8 Discover, Cu K_α_ radiation), with the x-ray beam being parallelized and monochromatized by an asymmetric Ge 2202-Bounce monochromator. A gate field was applied to bottom electrode while top electrode was grounded. The laser beam used for the x-ray diffraction measurements has a maximal power of 100 mW and a wavelength of 532 nm. The spot size of the laser beam is ~4 mm^2^, focusing on the regions where x-ray was reflected. All x-ray spectra were acquired at ambient temperature.

## Additional Information

**How to cite this article**: Li, Y. *et al.* Evidence for lattice-polarization-enhanced field effects at the SrTiO_3_-based heterointerface. *Sci. Rep.* **6**, 22418; doi: 10.1038/srep22418 (2016).

## Supplementary Material

Supplementary Information
