# Supplementary material for: Evidence for lattice-polarization-enhanced field effects at the SrTiO3-based heterointerface
Source: Sci Rep. 2016 Mar 1;6:22418. doi: 10.1038/srep22418 (PMC4772472; doi:10.1038/srep22418)
Supplement: Supplementary Information [file srep22418-s1.pdf]

Supplementary information for

Evidence for lattice-polarization-enhanced field effects at the $SrTiO_3$-based heterointerface

Ying Li[1], Hongrui Zhang[1], Yu Lei[1], Y. Z. Chen[2], N. Pryds[2], Baogen Shen[1], and Jirong Sun[1]

[1] Beijing National Laboratory for Condensed Matter Physics and Institute of Physics, Chinese Academy of Sciences, Beijing 100190, P. R. China
[2] Department of Energy Conversion and Storage, Technical University of Denmark, Risø Campus, 4000 Roskilde, Denmark

E-mail: *jrsun@iphy.ac.cn

Keywords: Lattice polarization, gating effect, combined electrical and optical stimuli, photo-excitation, oxygen vacancies

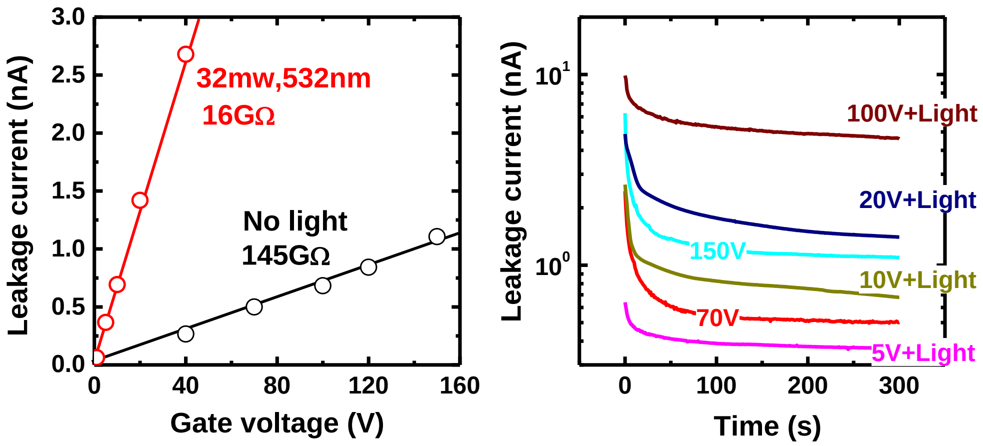

Supplementary Figure 1 Leakage current as a function of gate voltage. The deduced resistance of STO is ~145 GΩwithout illumination and ~16 GΩ when illuminated. The leakage current experiences a smooth decrease upon the application of gate field (right panel), due to polarization relaxation and charge trapping. Leakage current obtained under the gate field of opposite polarity is essentially the same. We note that in all cases, the leakage current was much lower than the applied in-plane current for resistance measurement, 1 μA.

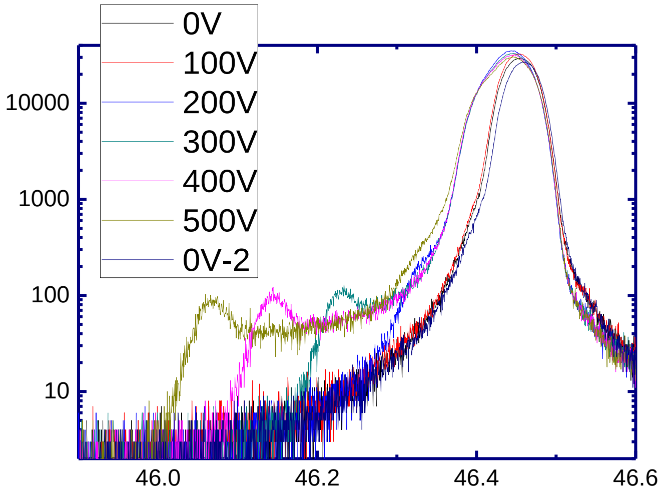

Supplementary Figure 2 X-ray diffraction patterns of a-LAO/STO under different gate voltages, recorded in a light of 100 mW (λ=532 nm). Out-of-plane lattice expansion takes place above the V_G of 200 V.

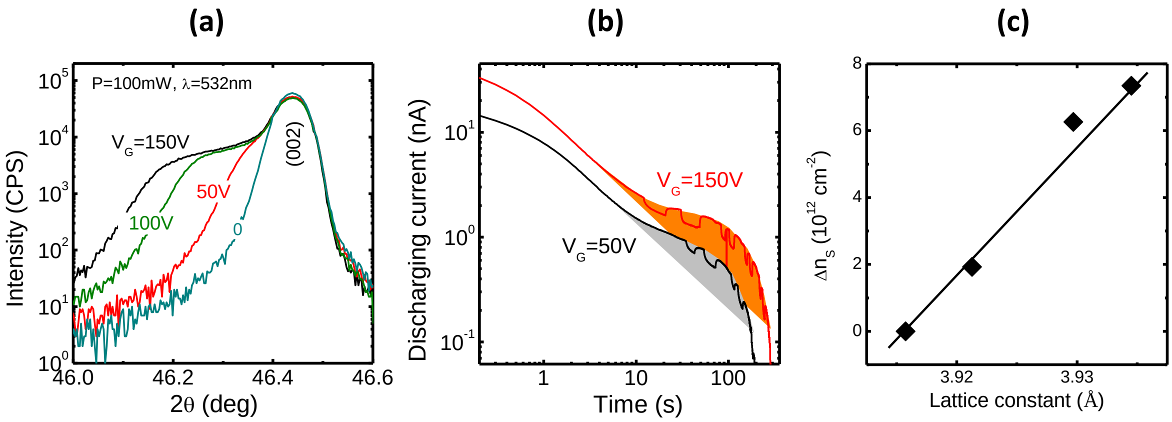

Supplementary Figure 3 (a) XRD patterns of the 002 reflections of STO, measured 10-min after the application of different gate voltage in a light of 100 mW (λ=532 nm). Different voltage produces different lattice expansion. (b) Discharging currents recorded after the removal of gate voltage. Valleys in the curve correspond to the on/off operation of the x-ray. (c) A relation between lattice expansion and discharged charges, obtained by integrating the transient current in the shaded area of (b). All measurements were performed at room temperature.

Supplementary Note 1 As a supplement, we would like to point out the difference between the abnormal lattice polarization and the ordinary electrical polarization of STO. As well known, a gate field will cause an electrical polarization of STO of the form of $P=\varepsilon_0(\varepsilon-1)E$, yielding the normal gating effect (the capacitive effect), where P and E are electrical polarization and electrical field, respectively. P is ~5.3×10^{-8} C/cm^2 under a gate voltage of 100 V at the room temperature. When lattice deformation occurs under the combined effects of gate field and photo excitation, a non-coincidence of the anionic and ionic centres may take place, causing lattice polarization. According to the above analysis, this effect is much stronger than the ordinary electrical polarization, $P=1.6\times10^{-6}$ C/cm^2 at the room temperature.

In general, the normalized sheet resistance change can be expressed as

$$R_S(-V_G)/R_S(V_G=0) = \left[1-\frac{\Delta n_S}{n_S}-\frac{\Delta\mu}{\mu}+\left(\frac{\Delta n_S}{n_S}\right)\left(\frac{\Delta\mu}{\mu}\right)\right]^{-1} \quad (1)$$

$$R_S(V_G)/R_S(V_G=0) = 1+\frac{\Delta n_S}{n_S}+\frac{\Delta\mu}{\mu}+\left(\frac{\Delta n_S}{n_S}\right)\left(\frac{\Delta\mu}{\mu}\right) \quad (2)$$

where Δn_S is gating effect-caused carrier density change, $\Delta\mu$ is the mobility variation corresponding to Δn_S. It is easy to prove that $R_S(-|V_G|)/R_S(0) \approx [\delta(1-\Delta\mu/\mu)]^{-1}$ when setting $\Delta n_S/n_S$ to $1-\delta$, where δ is a value much smaller than unity. Obviously, $R_S(-V_G)/R_S(V_G=0)$ will exhibit a continuous growth as $\Delta n_S/n_S$ and/or $\Delta\mu/\mu$ approach unity. This explains the high $R_S(-100V)/R_S(0)$ ratio observed in Figure 1b. In contrast, $R_S(|V_G|)/R_S(V_G=0)$ has the form of $2(1+\Delta\mu/\mu)$, i.e., its value is finite when $\delta\rightarrow0$. This analysis explains why $R_S(V_G)/R_S(V_G=0)$ is different under positive and negative V_Gs.

Supplementary Note 2 It is slightly complex to determine the lattice constant of the deformed layer of STO because of the absence of peak splitting in XRD spectra. We found that the XRD pattern can be well fitted by a single Gaussian function after removing gate voltage for more than 250 s (Fig. R1). We then safely say that in this case STO has completely returned to unstrained state. Comparing this XRD pattern (red curves in Fig. R2) with that recorded at earlier time (black curves in Fig. R2), we can clearly see the difference between these two spectra: Due to the appearance of a diffraction shoulder, the left side of the earlier pattern shows a left expansion, i.e., a diffraction shoulder appears about Δt time's earlier than the main 002 peak (Fig. R2). Without changing its general time dependence, we tentatively define $2\theta+\Delta$ as the peak position for deformed layer, where 2θ is the 002 peak position of unstrained STO and Δ is the low angle shift due to lattice expansion ($\Delta=\Delta t$*scanning speed, scanning speed=0.0124°/s). We estimated Δt along a dashed line around which the left sides of the two XRD patterns are nearly parallel with each other.

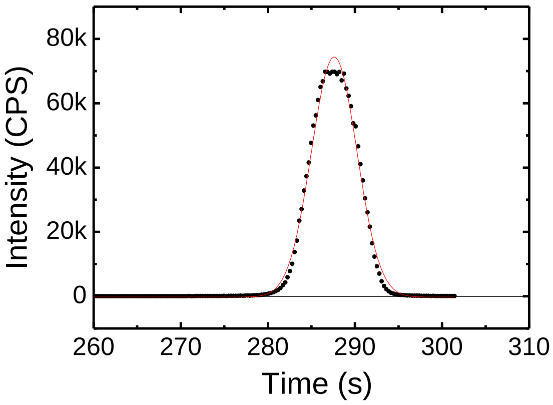

Fig. R1 x-ray diffraction pattern along time axis (scanning speed=0.0124°/s). In this case STO has returned to the unstrained state. Symbols=experiment results. Solid line is the result of curve fitting.

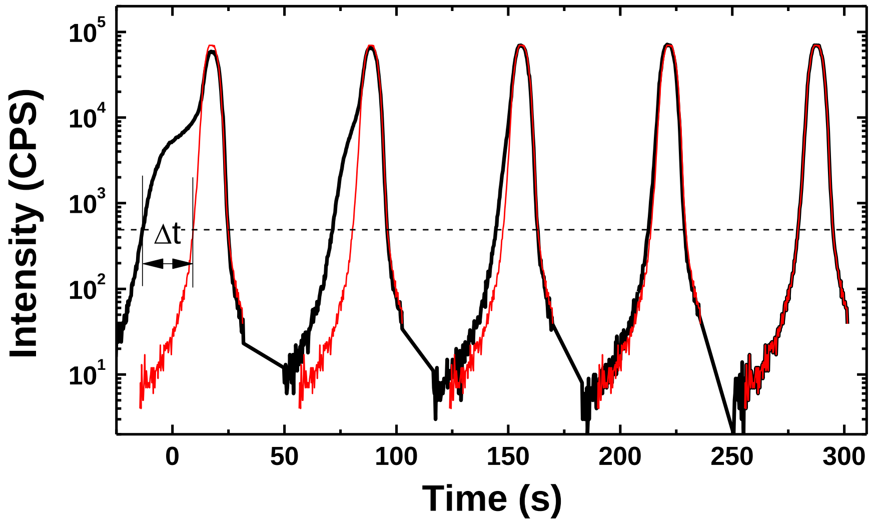

Fig. R2 A comparison of the x-ray diffraction patterns recorded at different time (black curves) with that shown in the Fig. R1 (red curve). For a clear comparison, the red curve has been rigidly shifted along the time axis. Dashed line marks the position where Δt is estimated, around which the left edges of the black and red curves are nearly parallel with each other.
